# Supplementary material for: Histopathology based AI model predicts anti-angiogenic therapy response in renal cancer clinical trial
Source: Nat Commun. 2025 Mar 17;16:2610. doi: 10.1038/s41467-025-57717-6 (PMC11914575; doi:10.1038/s41467-025-57717-6)
Supplement: Supplementary file 1 — Supplementary Information [file 41467_2025_57717_MOESM1_ESM.pdf]

# Supplementary Figures & Tables

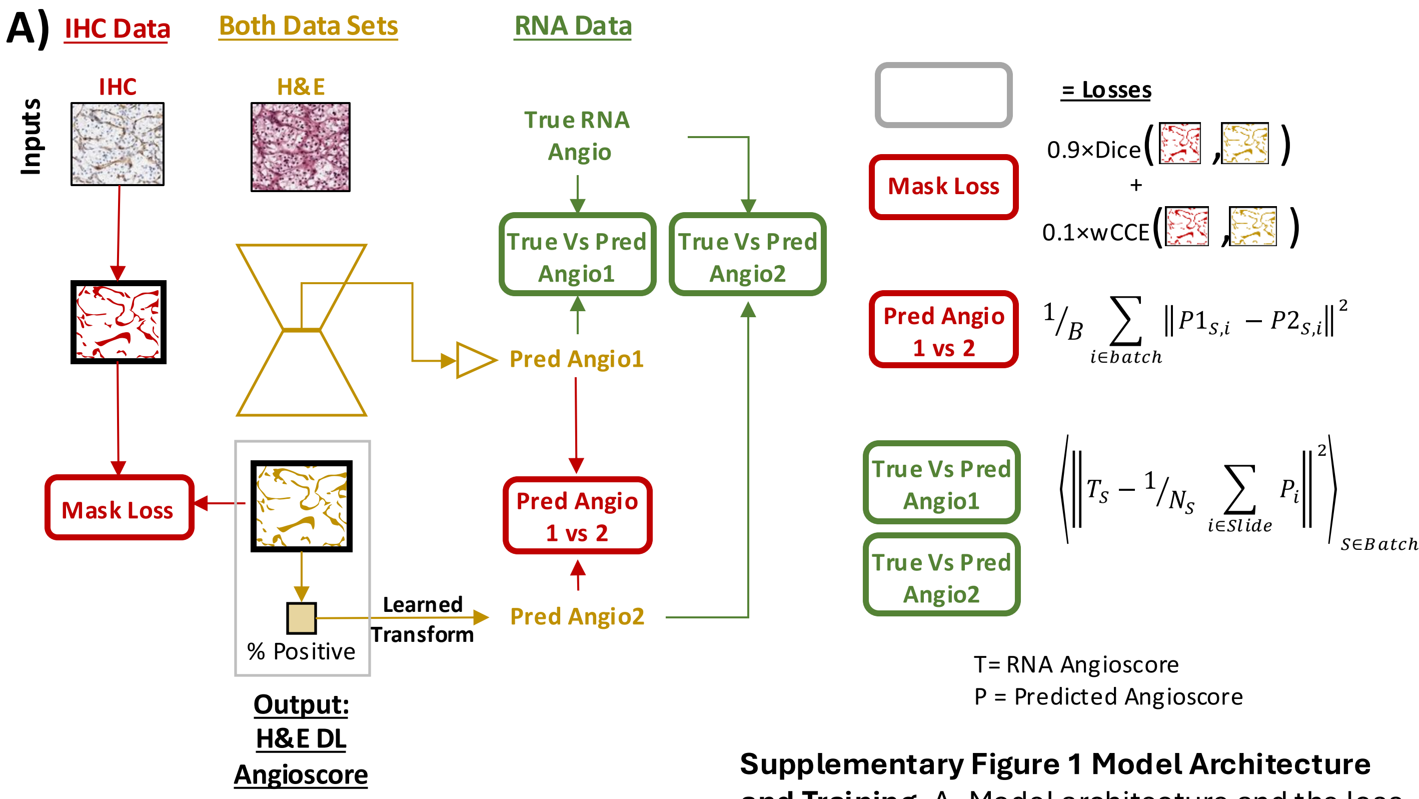


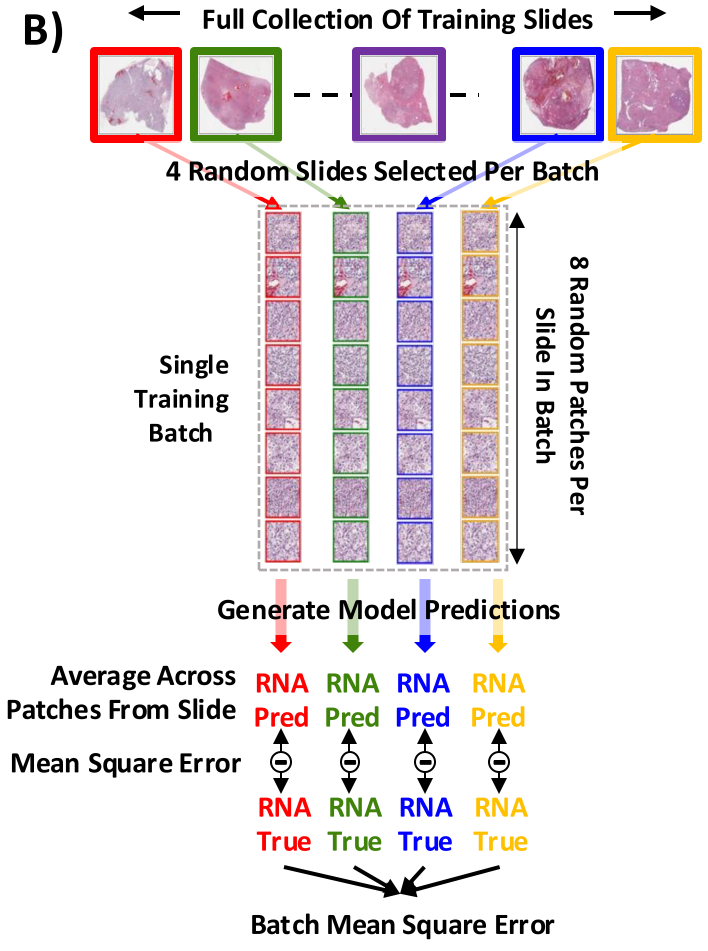


**Supplementary Figure 1 Model Architecture and Training.** A. Model architecture and the loss functions used for training the model. The model is a multi-task model that learns both regression (Angioscore prediction) and segmentation (vascular mask) from two different datasets. This mixed model was built to overcome a key challenge that each dataset only has ground truth for a single task, and thus during training we used distinct loss functions depending on the dataset from which an input patch is derived. RNA dataset from TCGA (green) produces H&E patches with true RNA score for training the regression arm, while an internal IHC dataset (red) of paired H&E and CD31 vascular masks are used for training the segmentation arm. Loss functions and network components are colored by the dataset on which they are applied with yellow denoting common to both data sets. A combination of Dice and weighted cross entropy loss was used for training the segmentation arm. Batch mean square error was used for training the regression arm. B. Illustration of patch sampling strategy for training the angioscore arm of the model. Batches comprise 32 patches, obtained by random selection of 4 training slides (N=4), followed by random sampling of 8 patches per slide ($N_S$=8)

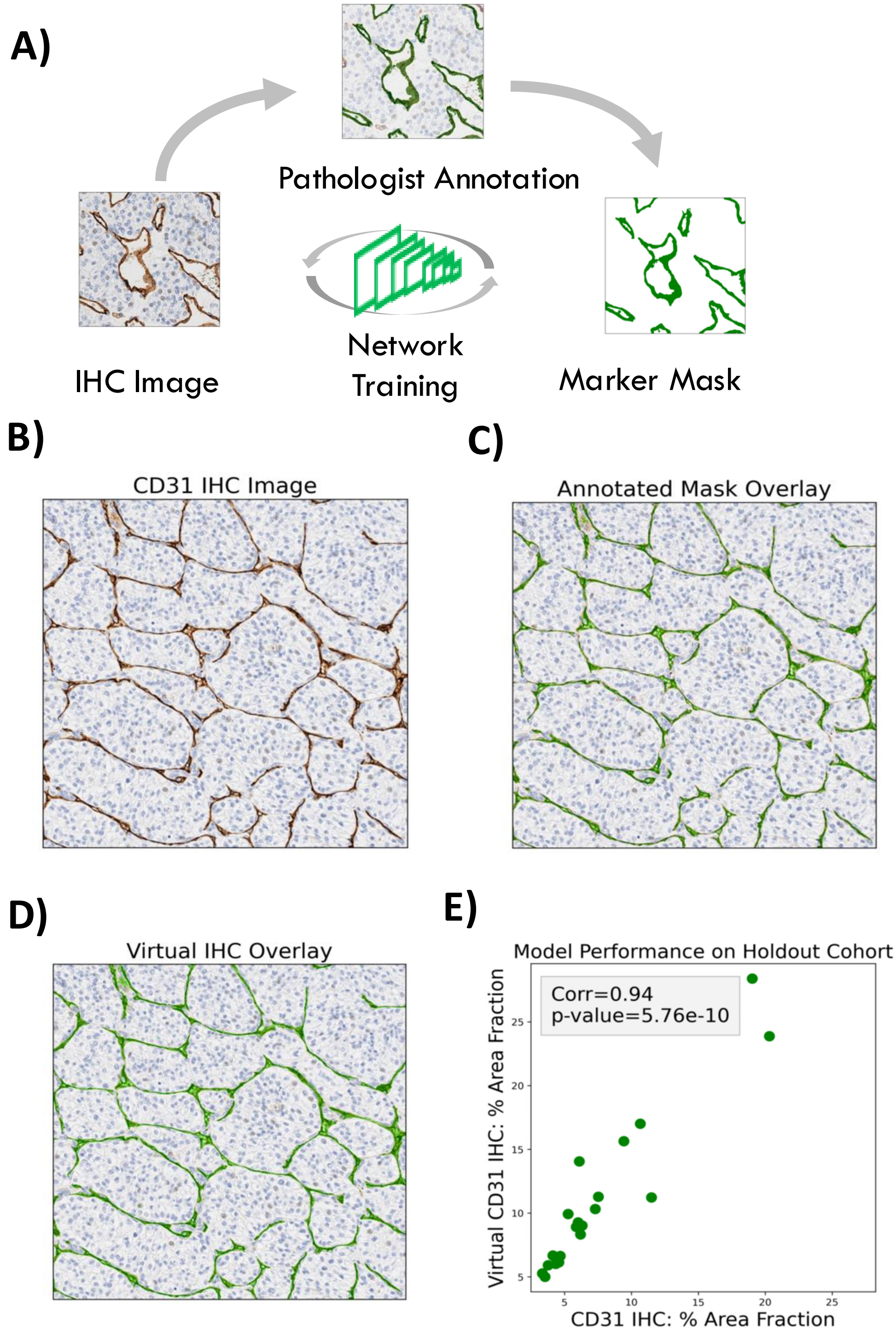


**Supplementary Figure 2. Schematic and sampling of results illustrating CD31 IHC segmentation model development. A.)** Model development. Patches were generated from CD31 stained slides (IHC Image). Corresponding masks were generated from pathologist annotation. The image and the mask pairs are used for training a UNet for determining segmentation masks, which in turn are used as ground truth masks for H&E segmentation model. **B.)**, **C.)**, and **D.)** show a sample CD31 image, annotated mask overlaid on the image, and model prediction of the mask, respectively. Precision, recall, and F1 score metrics are:0.67, 1.0, and 0.8. **E.)** Correlation between the model-predicted area fraction and true area fraction from annotated masks from a sampling of patches from test set. Source data are provided as a Source Data file. P-values are calculated using 2-sided Spearman rank correlation test.

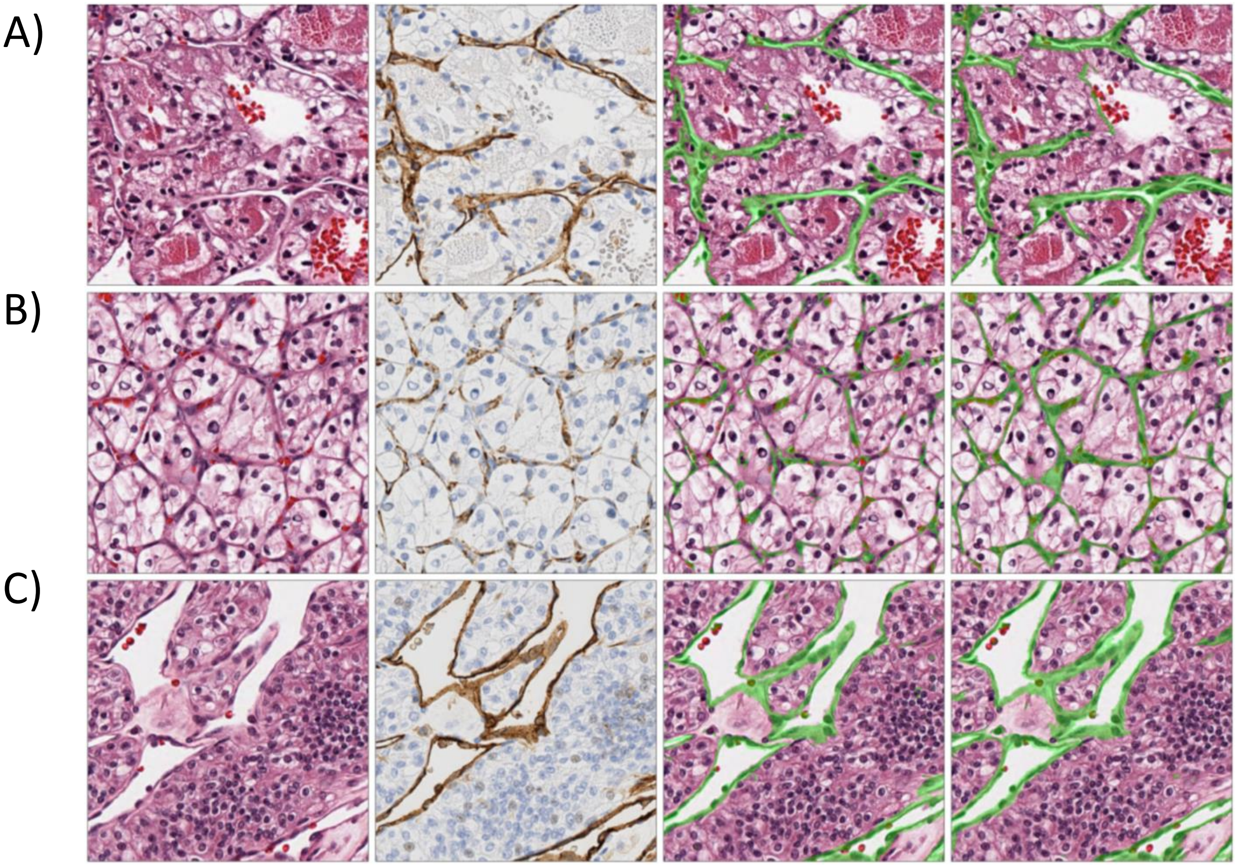


**Supplementary Figure. 3. Sample comparisons of vascular mask predictions to ground truth based on CD31 in the held-out portion of the UTSW CD31 training dataset**. Shown are representative H&E-stained image (first column), the matching IHC-stained image obtained from registration (second column), the vascular mask predicted by the IHC segmentation model using the IHC image from the second column (third column), and the mask the prediction from the mixed model (fourth column). Figures A-C show a sampling of images from the held-out set. Figure B illustrates an example of faint CD31 IHC stain with nonreactive vascular network as shown in 3rd column. The H&E model predictions compensate for the short coming in staining (column 4). However, since we are using IHC predictions as ground truth, this results in over prediction (lower precision).

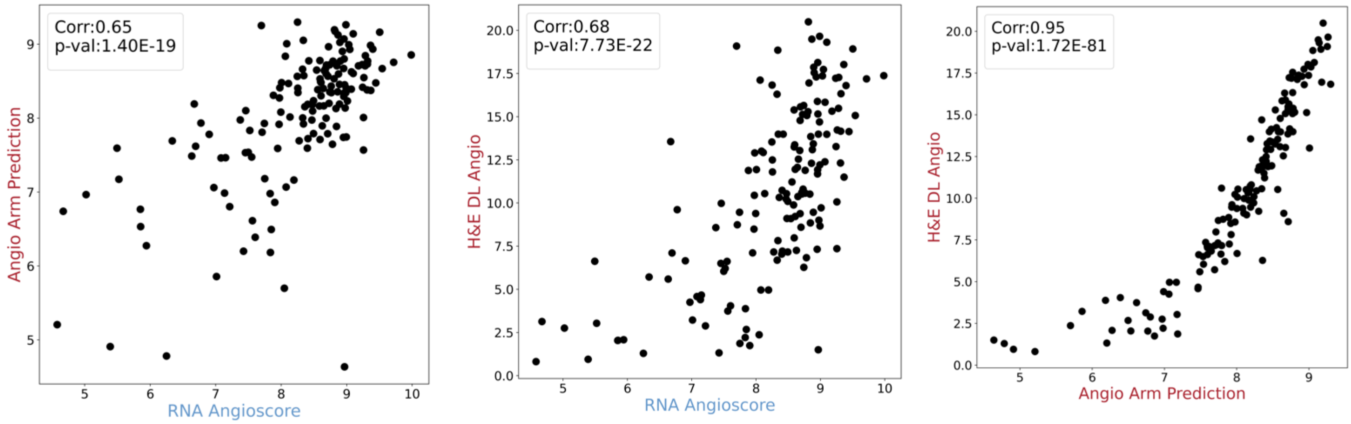


**Supplementary Figure 4. Comparison of the two Angioscore prediction arms of the mixed model on held out TCGA data.** The mixed model predicts the Angioscore in two ways: one based on a regression model (denoted as Angio Arm Prediction) that directly predicts the Angioscore, and the other based on the percentage of positive pixels in the vascular segmentation mask (denoted as H&E DL Angio). During training we encourage these two outputs to agree, and here we compare the outputs of these two arms to the ground truth RNA Angioscore in the TCGA held-out set. Each point represents a single RNA sample and the model predictions across all patches from that sample were averaged. Note that the Pearson correlation between the two arms Is high (0.95). P-values are calculated using two-sided Spearman rank correlation test (N=154 samples). Source data are provided as a Source Data file.

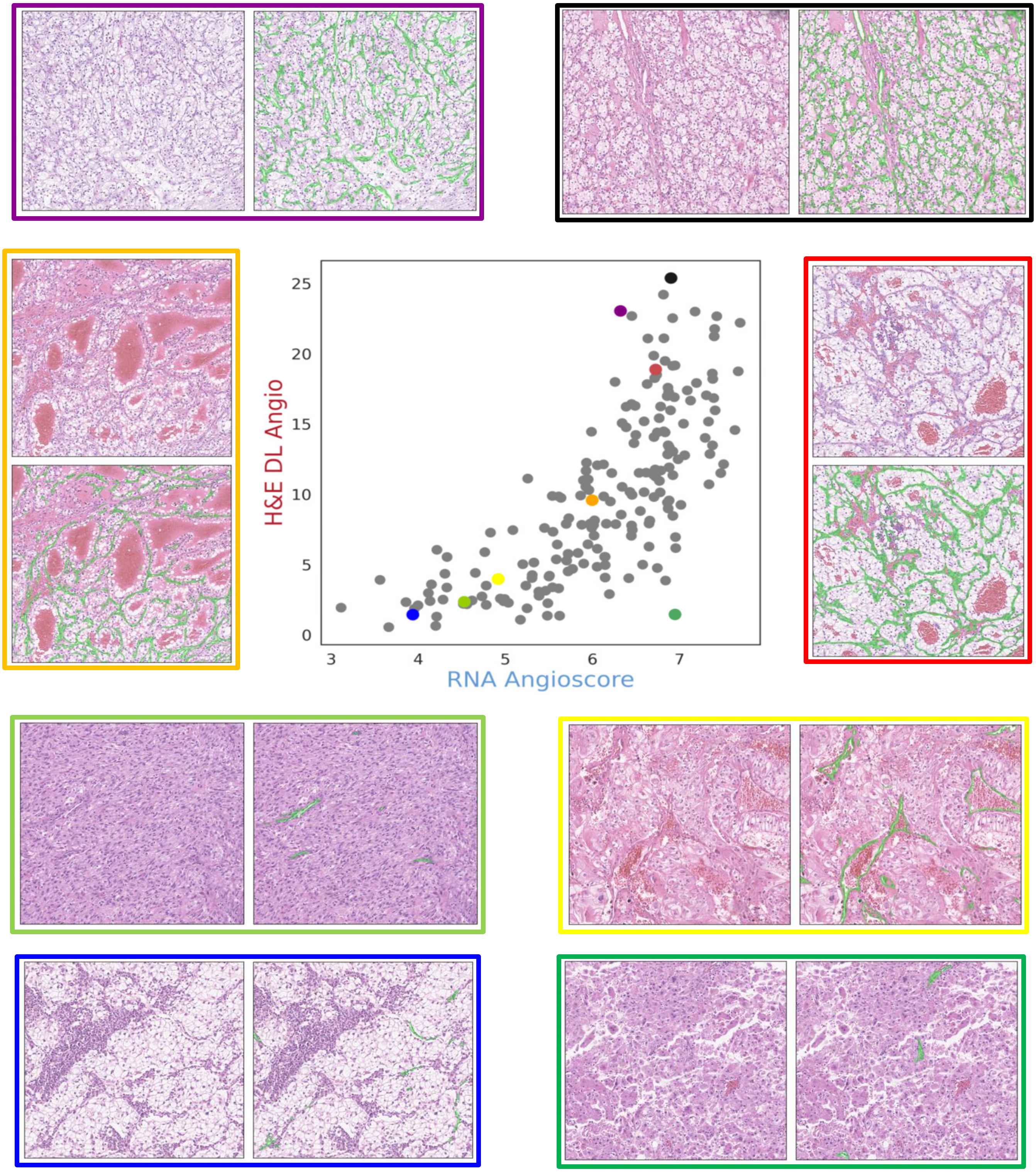


**Supplementary Figure 5. Sample images and predicted vascular masks from UTSEQ dataset.** The scatter plot at the center of the figure is the same as Fig. 2B and reproduced here to show the location of patches on the plot. Representative images illustrating a range of vascular architecture patterns and Angioscores is shown starting anti-clock direction from the top right: small nest (black), small nest (maroon), bleeding follicles (orange), solid sarcomatoid (light green), immune-rich trabecular pattern (blue), pseudo-papillary (green), solid-rhabdoid (yellow), and alveolar (red). Note: prediction for the papillary sample (green) is consistent with what one would expect based on the image (i.e., very little vasculature) but the sample has one of the highest measured RNA Angioscore. There were large blood vessels near the tumor (not shown), and the tissue used for RNA analysis likely captured these blood vessels. Source data are provided as a Source Data file.

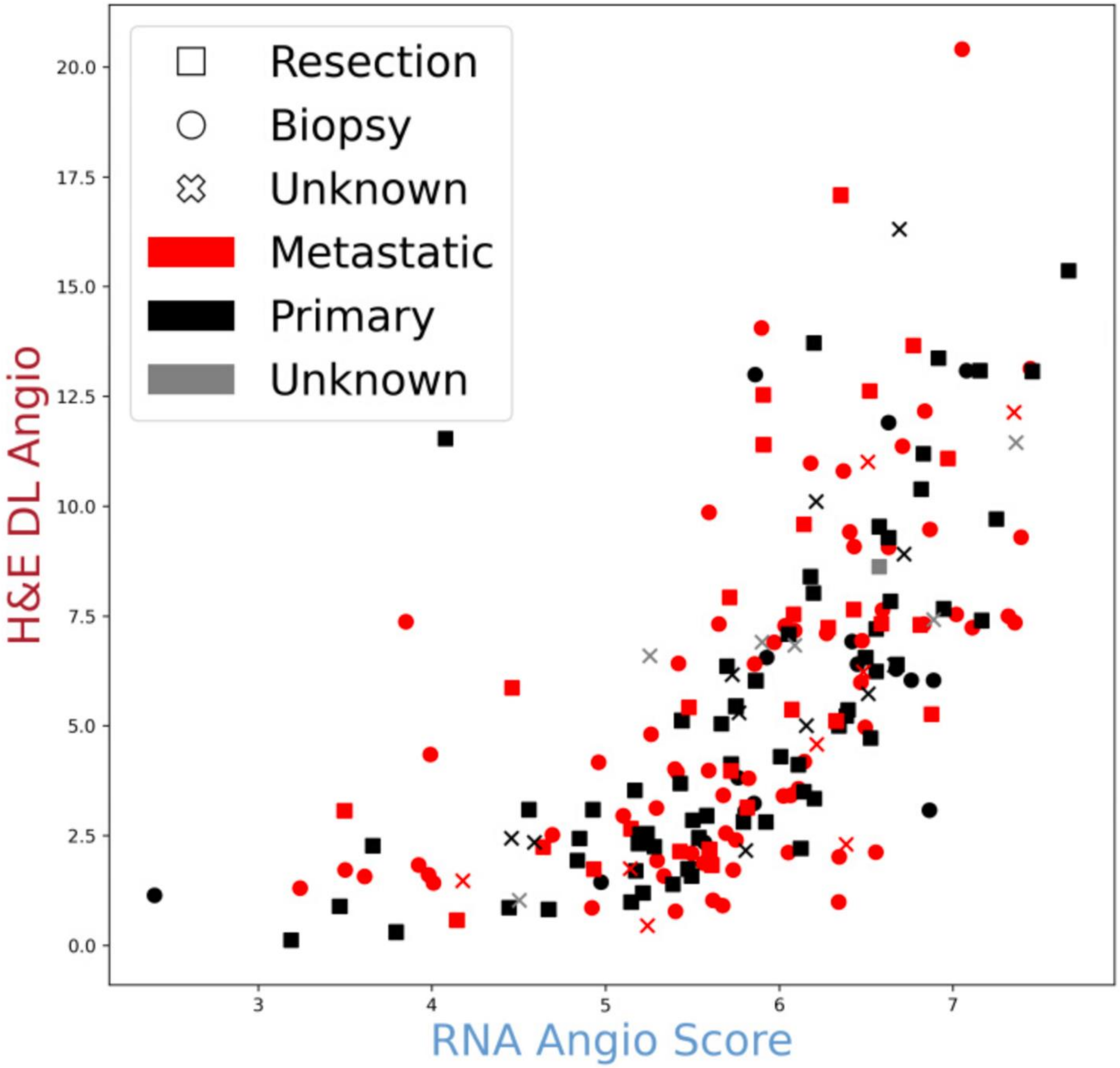


**Supplementary Figure 6. Effect of tumor site and sample extraction procedure on model performance for the IMmotion 150 dataset (site and procedure overlayed on Fig. 2C).** Extraction site (primary, metastatic, not available) data is represented in marker color while extraction type (resection, biopsy or not available) is represented in marker shape. Source data are provided as a Source Data file.

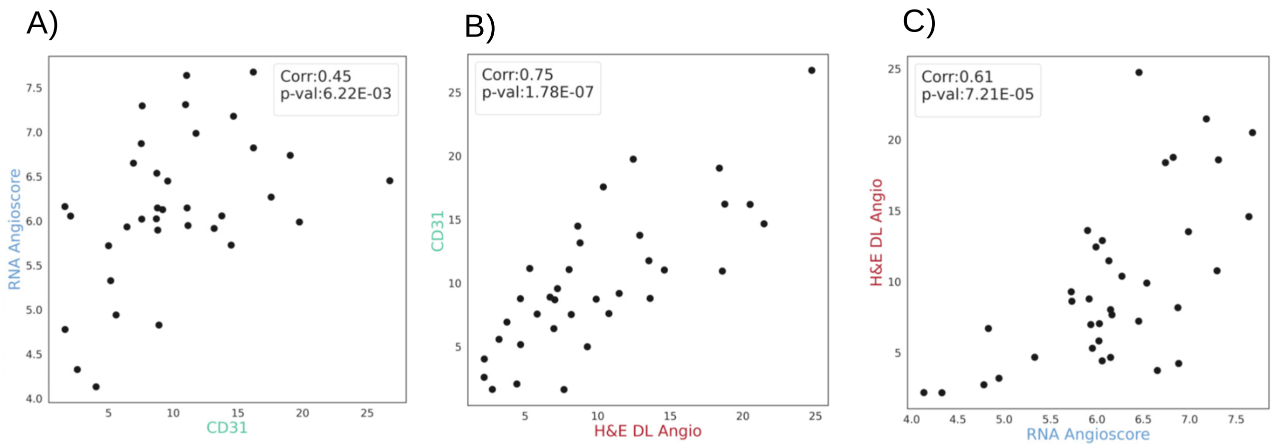


**Supplementary Figure 7**. **Comparison of model prediction to CD31 IHC in UTSeq cohort**. For a subset of cases (n=35) in the UTSeq cohort we performed CD31 IHC on a serial section adjacent to the H&E-stained section used to predict the H&E DL Angioscore. For each sample we compare the true RNA Angioscore (blue), H&E DL Angioscore (red) and the percentage of CD31 positive area (green). **A.)** CD31 vs RNA Angioscore. **B.)** CD31 vs H&E DL Angioscore. C.) H&E DL Angioscore vs RNA Angioscore. Note: Unlike in the main figures (where we average predictions from H&E images at the top and bottom of each punch), here we only make use of a single "bottom" H&E slide which is closest to the single CD31 section generated for each slide. P-values are calculated using two-sided Spearman rank correlation test. Source data are provided as a Source Data file.

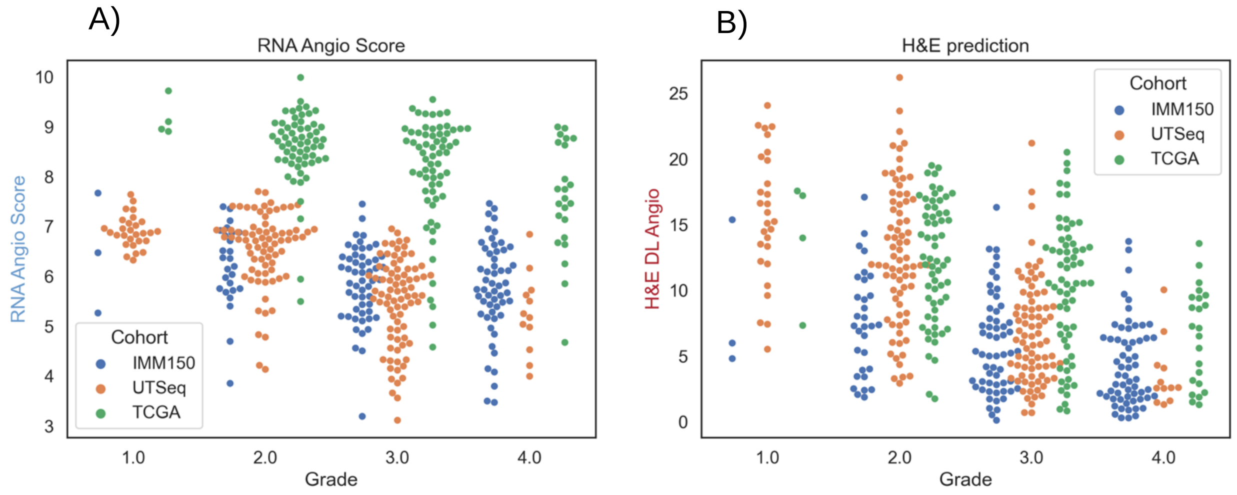


**Supplementary Figure 8**. **Effect of batch/cohort in Angioscore prediction from RNA and H&E DL model.** This figure illustrates the Angioscores for all 3 cohorts (TCGA held out data: green, UTSeq: orange, and IMmotion 150: blue) stratified by nuclear grade. **A).** Based on RNA Angioscore. **B).** Based on H&E DL Angioscore. Source data are provided as a Source Data file.

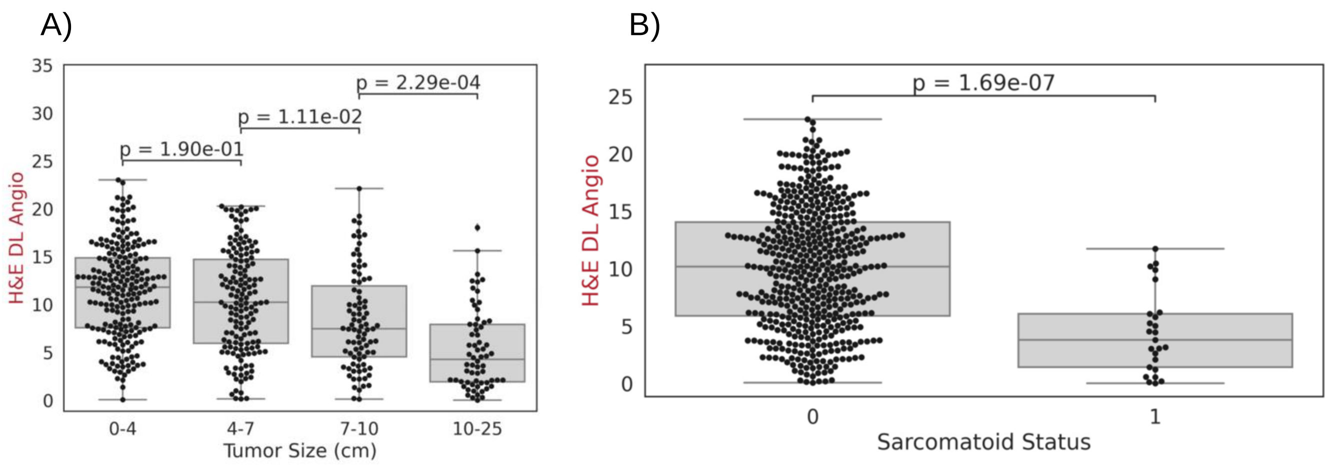


**Supplementary Figure 9**. **Relationship between H&E DL Angioscore and other prognostic variables.** The H&E DL Angioscore model was applied to independent UTSW tissue microarray cohorts and its output was compared to known prognostic variables including on **A)** Tumor size (N[0-4]=216,N[4-7]=153,N[7-10]=87,N[10-25]=64 **B)** Presence/Absence of Sarcomatoid tissue in the sample N[0]=495, N[1]=25. P-values are calculated using Mann-Whitney U test, a nonparametric two-sided test. Box plots show median values with inter quartile ranges (IQR) and the whiskers show 1.5 times IQR values. Source data are provided as a Source Data file.

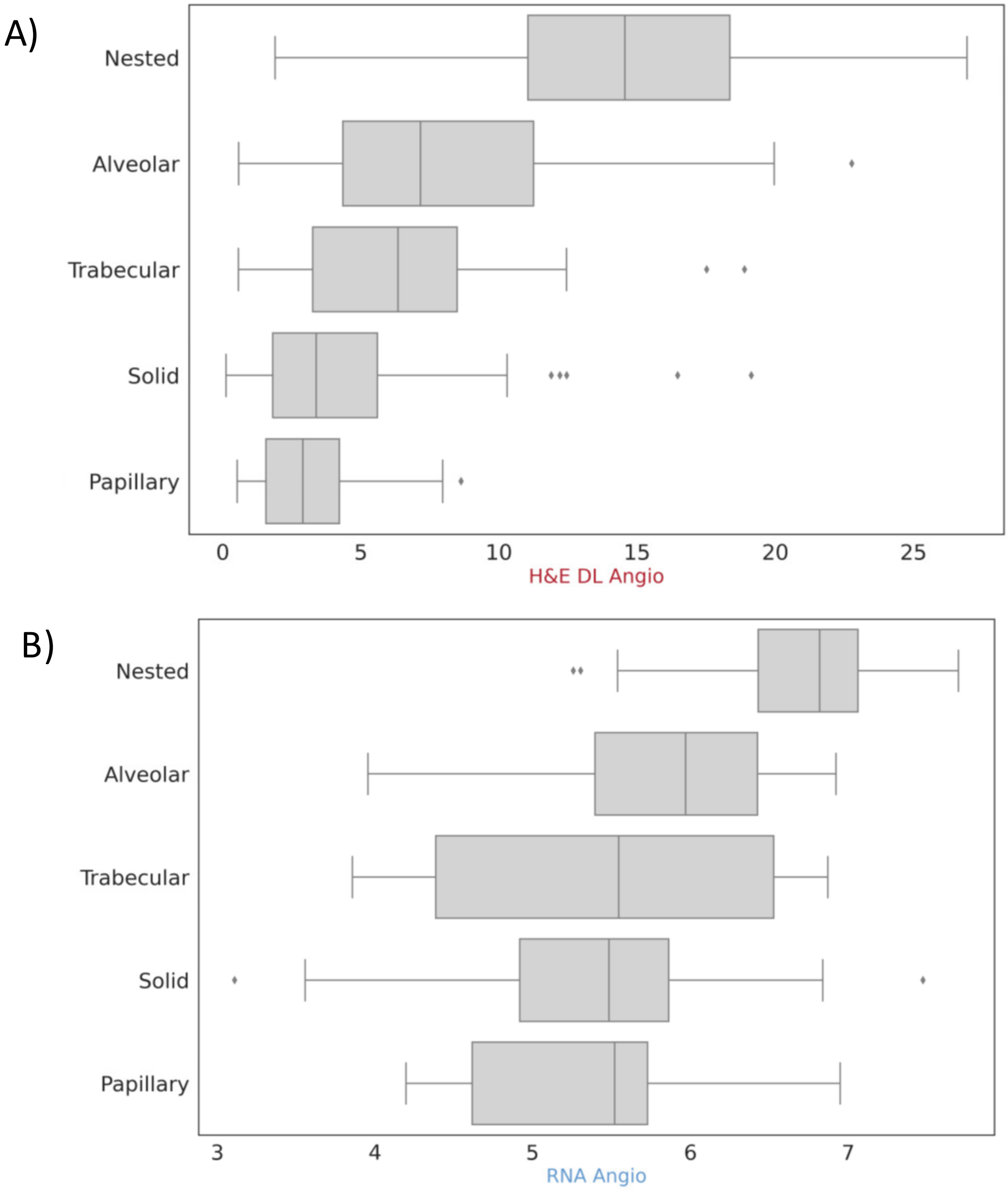


**Supplementary Figure 10. Relation between morphologic architecture patterns and H&E DL / RNA-based Angioscore.** Morphologic architectures (that are based on vascular patterns) were manually assessed by a trained pathologist (PK) for each sample (blinded to the Angioscores) in the UTSeq dataset and shown boxplots compare the Angioscores based on A) H&E DL and B) RNA angioscores. Box plots in A-C show median values with inter quartile ranges (IQR) and the whiskers show 1.5 times IQR values. Source data are provided as a Source Data file.

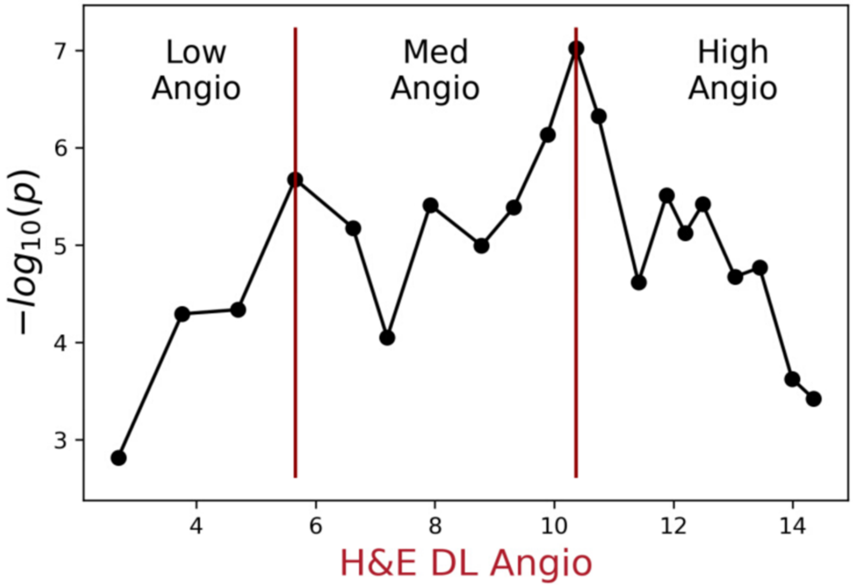


**Supplementary Figure 11. Choice of H&E DL Angio cutoff from overall survival analysis on TCGA data.** In the TCGA data, we considered a range of 20 equally spaced H&E DL Angio Score (x-axis) cutoffs for stratifying patients into high angioscore and low angioscore. For each, a P-value (y-axis transformed with negative logarithm) was calculated from two-sided non-parametric log rank test. The two peaks form the basis of our splitting the data into three groups. Source data are provided as a Source Data file.

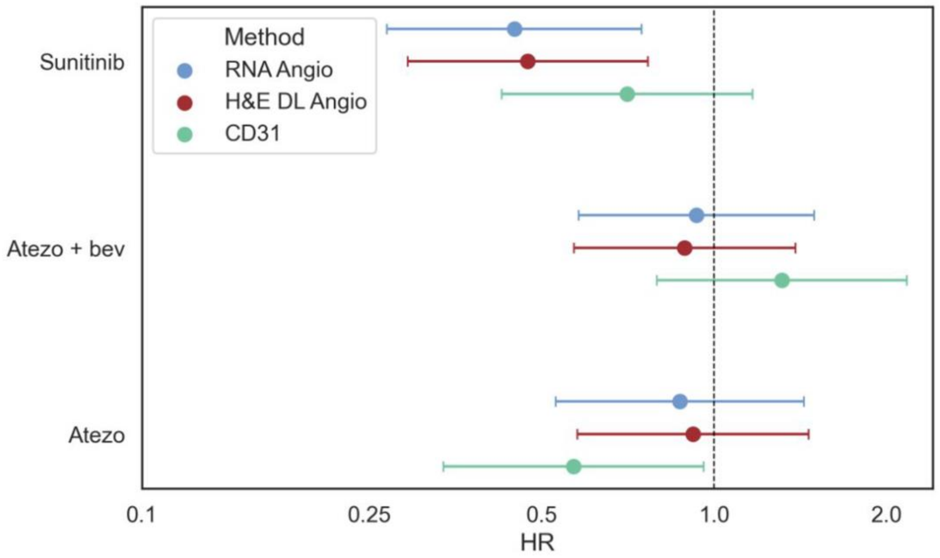


**Supplementary Figure 12. Comparison of hazard ratios for different measures of Angiogenesis across the 3 arms of IMmotion150 dataset.** Patients from all three arms (Sunitinib, Atezolizumab, and combination of Atezolizumab and bevacizumab) of IMmotion 150 study were assigned to low/high angiogenesis groups based on RNA, H&E and CD31 and separate Cox-proportional hazard models were used to determine hazard ratios for each combination of assay and arm. Source data are provided as a Source Data file.

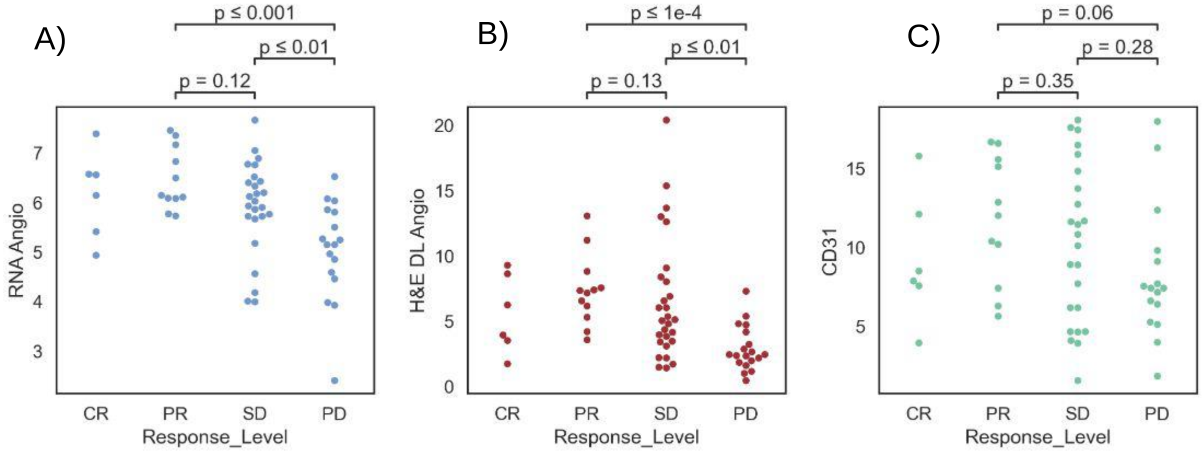


**Supplementary Figure 13. Relationship between Sunitinib response in IMmotion150 and different Angiogenesis measures.** Comparison of Angiogenesis levels in patients at different levels of response to Sunitinib in the IMmotion150 cohort based on **A.)** RNA Angioscore **B.)** H&E DL Angioscore and **C.)** CD31. CR = complete response, PR = partial response, SD = stable disease, PD = progressive disease. P-values are calculated using Mann-Whitney U test, a non-parametric two- sided test.

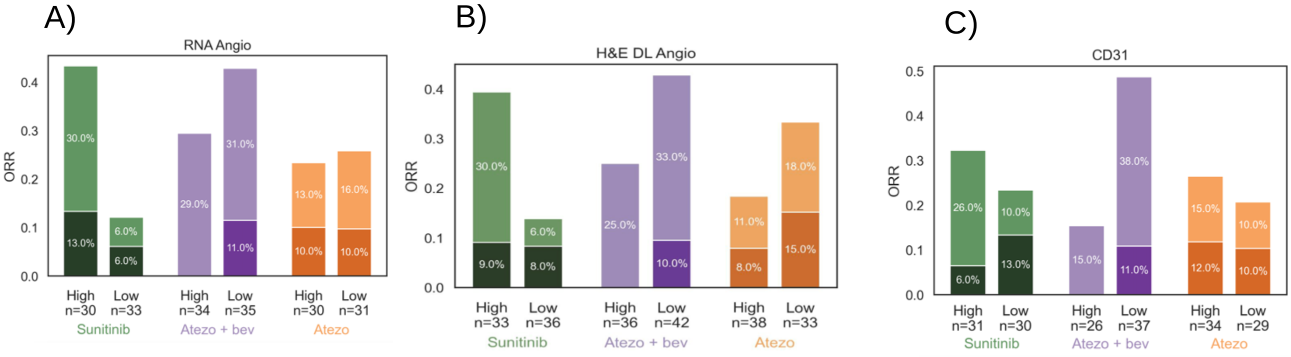


**Supplementary Figure 14. Relationship between treatment response level and Low/High Angiogenesis for different arms of IMmotion 150 dataset.** High and Low populations are determined by median Angioscores. Each treatment arm is color coded for the 3 drug treatment arms with darker shade representing complete responders while lighter shade represents partial responders. **A.)** Response to treatment as measured by RNA Angioscore. **B.)** Response to treatment as measured by H&E DL Angioscore. **C.)** Response to treatment as measured by CD31. (Atezo: atezolizumab; bev: bevacizumab, PR: partial response, CR: complete response). Source data are provided as a Source Data file.

**Supplementary Table 1. Datasets used for this work (OS: Overall Survival, TNT: Time to next treatment, PFS: Progression Free Survival)**

| Dataset | Number of Samples | Use | RNA | CD31 | Clinical | Survival |
|---|---|---|---|---|---|---|
| TCGA-KIRC (Patients) | 462-patients | Training /Holdout/Survival | ✓ | | ✓ | OS |
| UTSW-CD31 Re-Stain | 17 slides | Training/Holdout | | Image | | |
| UTSW - UTSeq | 161 samples | Validate Angio/ Vascular prediction | ✓ | Subset with Image | | |
| UTSW-TMA | 811 TMA punches | Explore Angio to Clinical Correlates | | | ✓ | OS |
| UTSW – TKI | 145 patients | Validate Response Prediction | | | | TNT |
| IMmotion 150 | 226 slides | Validate Angio and Response Prediction | ✓ | Score | ✓ | PFS |

**Supplementary Table 2. Cross validation results on Spearman correlations of H&E DL Angio score predictions with RNA Angioscore**

| Dataset | Fold 1 | Fold 2 | Fold 3 | Mean | Sigma |
|---|---|---|---|---|---|
| TCGA-CV | 0.68 | 0.53 | 0.63 | 0.61 | 0.062 |
| UTSW-UTSeq | 0.77 | 0.76 | 0.74 | 0.76 | 0.012 |
| IMmotion-150 | 0.73 | 0.73 | 0.72 | 0.72 | 0.005 |

# Supplementary Table 3. Summary of Different Comparator Models Used in Study

| Model | Network Part Trained | Unit of Prediction | Patches per slide per batch | Backbone | Loss | RNA Corr TCGA | RNA Corr UTSeq | RNA Corr IMM150 |
|---|---|---|---|---|---|---|---|---|
| Mixed (Angio+CD31) | End-to-End | Patch + pixel | 8 | UNet ResNet 18 Encoder | Batch MSE + (CCE+ Dice) | 0.68 | 0.77 | 0.73 |
| Angio only | End-to-End | Patch | 8 | ResNet 18 | Batch MSE | 0.63 | 0.59 | 0.52 |
| Angio only | Last Conv + Fc layer trained | Patch | 8 | ResNet 18 | Batch MSE | 0.63 | 0.59 | 0.49 |
| Angio only | Fc Layer trained | Patch | 8 | ResNet 18 | Batch MSE | 0.54 | 0.65 | 0.54 |
| MIL Regression CAMIL | Post Feature Extraction Network | Patch | 1500 | Custom | MIL Loss | 0.54 | 0.67 | 0.55 |
| CD31 Only | End-to-End | Pixel | 16* | UNet with Resnet 18 encoder | CCE + Dice | 0.52 | 0.67 | 0.54 |

- Randomly selected from the entire training set
- All correlations are Spearman

**Supplementary Table 4. Overall Survival, Treatment Response Data for Various Cohorts** (p-values are calculated using two-sided non-parametric log rank test. )

| Dataset | Threshold H&E DL Angio | P value | HR | 95% HR | Nlow | Nhigh | C-index |
|---|---|---|---|---|---|---|---|
| TCGA-OS | 5.66 | 2.11e-6 | 2.24 | 1.6 – 3.13 | 98 | 364 | 0.656 |
| TCGA-OS | 10.37* | 9.5e-8 | 2.42 | 1.75-3.36 | 216 | 246 | 0.656 |
| TMA-OS | 5.42* | 2.0e-11 | 4.62 | 2.95-7.22 | 129 | 391 | 0.748 |
| TMA-OS | 5.66 | 5.0e-11 | 4.54 | 2.89-7.11 | 135 | 385 | 0.748 |
| UTSW-TKI | 2.34* | 8.7e-5 | 0.41 | 0.26-0.64 | 31 | 114 | 0.597 |
| UTSW-TKI | 5.66 | 1.23e-2 | 0.64 | 0.45-0.91 | 65 | 80 | 0.597 |
| IMmotion-150-TKI | 5.66 | 2.45e-4 | 0.39 | 0.23-0.64 | 45 | 27 | 0.66 |
| IMmotion-150-TKI | 4.61** | 2.36e-3 | 0.45 | 0.27-0.75 | 35 | 37 | 0.66 |

*Best threshold **Median Value

**Supplementary Table 5. Comparison of C-index values of various risk stratification metrics against progression free survival analysis for Sunitinib treatment arm of IMmotion 150 dataset**

| Parameter | C-index |
|---|---|
| H&E DL Angio | 0.659 |
| RNA Angio | 0.671 |
| CD31 | 0.549 |
| MSKCC | 0.531 |
| Grade | 0.53 |
| MTZRGR | 0.577 |

**Supplementary Table 6. Comparison of Spearman correlations from different outputs of the mixed model on TCGA holdout set**

| Epoch | Angio Arm | Mask Angio Arm | Correlation with positive Fraction from Segmentation mask |
|---|---|---|---|
| 1 | 0.61 | 0.58 | 0.59 |
| 2 | 0.63 | 0.60 | 0.62 |
| 3 | 0.66 | 0.62 | 0.63 |
| 4 | 0.64 | 0.62 | 0.63 |
| 5 | 0.63 | 0.62 | 0.64 |
| 6 | 0.64 | 0.63 | 0.64 |
| 7 | 0.61 | 0.63 | 0.65 |
| 8 | 0.65 | 0.66 | 0.68 |
| 9 | 0.61 | 0.63 | 0.66 |
| 10 | 0.63 | 0.63 | 0.65 |
